# Long-term effect of intravenous antimicrobial use in a pharmacist-led ASP at a small Japanese acute care hospital

**DOI:** 10.1017/ash.2024.149

**Published:** 2024-09-16

**Authors:** Yasuaki Tagashira, Yasuhiro Sasaki

**Affiliations:** Tokyo Medical and Dental University; Tokyo Metropolitan Tama Medical Center

## Abstract

**Background:** Antimicrobial resistance (AMR) remains a crucial, healthcare issue for which many countries have devised a national action plan. In Japan as well, antimicrobial stewardship programs (ASP) are being implemented in acute care hospitals under this policy framework. Clinical pharmacists play a central role in ASP, often jointly with infectious disease (ID) physicians. However, in Japan, a shortage of ID physicians has resulted in some ASP being led solely by pharmacists. While reports of the short-term effects of this situation are emerging, the long-term impact of pharmacist-led ASP is still largely unknown in Japan. The present study retrospectively examined the long-term effects of pharmacist-led ASP in a small, Japanese, acute care hospital. **Method:** The present study examined a pharmacist-led ASP in an acute care hospital (287 beds) in Japan which was launched in August 2015 and assessed the duration of therapy per 1000 patient-days (DOT) as the primary outcome by comparing the pre-intervention period (April 2013-July 2015) with the intervention period (August 2015-March 2023) using linear regression analysis. Additionally, segmented time-series analysis was conducted for each, additional intervention, and the impact of reduced activity due to the coronavirus disease 2019 (COVID-19) pandemic during the intervention. The DOT at the study center were compared with the national average of facilities implementing ASP. **Result:** While the DOT for all intravenous antimicrobials showed a slight increase on linear regression (r=0.01; P=0.1), the DOT of antipseudomonal intravenous antimicrobials significantly decreased (r=-0.027; P < 0 .01). Moreover, a significant reduction in DOT was observed immediately after the initiation of prospective review and feedback for carbapenems and daily prospective review and feedback for all intravenous antimicrobials (-3.2 and -2.4; P < 0 .001 for the intercept). An increase in DOT was observed during the COVID-19 pandemic-related reduction in activity time, and a rapid decline was observed upon the resumption of activities. Conversely, the average, nationwide DOT significantly increased for all intravenous antimicrobials as well as for antipseudomonal intravenous antimicrobials (r=0.02 and r=0.004; P < 0 .01) **Conclusion:** Sustaining an effective, pharmacist-led antimicrobial stewardship program led to a continual decrease in the DOT of antipseudomonal intravenous antimicrobials in a small, Japanese, acute care hospital despite a nationwide increase in their use following implementation of the national AMR action plan. Detailed analysis of pharmacists’ activities across multiple facilities is necessary to verify these effects.

